# Prostate Cancer Liver Metastasis: An Ominous Metastatic Site in Need of Distinct Management Strategies

**DOI:** 10.3390/jcm13030734

**Published:** 2024-01-27

**Authors:** Audrey Shiner, Rubens Copia Sperandio, Mahdi Naimi, Urban Emmenegger

**Affiliations:** 1 Division of Medical Oncology, Odette Cancer Centre, Sunnybrook Health Sciences Centre, Toronto, ON M4N 3M5, Canada; audrey.shiner@sri.utoronto.ca (A.S.); rubens.copiasperandio@sunnybrook.ca (R.C.S.); mahdi.naimi@sri.utoronto.ca (M.N.); 2 Sunnybrook Research Institute, Sunnybrook Health Sciences Centre, Toronto, ON M4N 3M5, Canada; 3 Institute of Medical Science, University of Toronto, Toronto, ON M5S 1A8, Canada; 4 Temerty Faculty of Medicine, University of Toronto, Toronto, ON M5S 1A8, Canada

**Keywords:** castration-resistant prostate cancer, liver metastasis, liver injury, risk factors

## Abstract

Prostate cancer liver metastasis (PCLM), seen in upwards of 25% of metastatic castration-resistant PC (mCRPC) patients, is the most lethal site of mCRPC with a median overall survival of 10–14 months. Despite its ominous prognosis and anticipated rise in incidence due to longer survival with contemporary therapy, PCLM is understudied. This review aims to summarize the existing literature regarding the risk factors associated with the development of PCLM, and to identify areas warranting further research. A literature search was conducted through Ovid MEDLINE from 2000 to March 2023. Relevant subject headings and text words were used to capture the following concepts: “Prostatic Neoplasms”, “Liver Neoplasms”, and “Neoplasm Metastasis”. Citation searching identified additional manuscripts. Forty-one studies were retained for detailed analysis. The clinical risk factors for visceral/liver metastasis included <70 years, ≥T3 tumor, N1 nodal stage, de novo metastasis, PSA >20 ng/mL, and a Gleason score >8. Additional risk factors comprised elevated serum AST, LDH or ALP, decreased Hb, genetic markers like RB1 and PTEN loss, PIK3CB and MYC amplification, as well as numerous PC treatments either acting directly or indirectly through inducing liver injury. Further research regarding predictive factors, early detection strategies, and targeted therapies for PCLM are critical for improving patient outcomes.

## 1. Introduction

Prostate cancer (PC) is the second leading cancer diagnosed among males worldwide [1]. While localized PC is highly curable, around 5–10% of patients in developed countries present with metastases at diagnosis, and around 20–30% develop metastases despite curative treatment attempts [2,3]. Metastatic PC, notably in its castration-resistant state (i.e., mCRPC), is an ultimately fatal condition, which accounted for more than 375,000 deaths globally in 2020 [1,4,5,6].

The first site of PC metastasis tends to be the lymph nodes adjacent to the prostate, followed by bone and distant lymph nodes [7]. Hence, most of the existing literature focuses on bone (~90% of mCRPC patients) and nodal (~50%) metastasis, whereas research on PC liver metastasis (PCLM) is limited [8,9,10,11,12]. This is highly problematic as the liver is the most lethal metastatic site of mCRPC, being associated with a median overall survival of 10 to 14 months [10,13,14,15,16]. In fact, the hazard ratio of death from PCLM is the highest compared to LM from other tumor types [17]. In addition, PC patients are often not routinely screened for visceral metastasis such as LM, and LM does not seem to respond as well to conventional therapies, such as hormonal or chemotherapy, that are successfully used to treat other metastatic sites of mCRPC [17,18,19,20]. While PC visceral metastasis in general renders an unfavourable prognosis, patients with LM have much worse outcomes than those with lung metastases, regardless of treatment [20,21].

While it has been reported in clinical series that 3–12% of patients with mCRPC have LM, the liver is a challenging metastatic site to study, as there is a great extent of initial metastatic dormancy [14,15,16,22,23]. Moreover, unlike bone metastasis, which tends to cause pain, early LM often does not present notable symptoms [12]. Thus, PCLM is often detected at an advanced stage with widespread liver involvement, or in some patients, may go undetected until death [10]. This is indicated in an autopsy study reporting 25% of PC patients having LM, suggesting that the true incidence of PCLM may be higher than reported in the clinical literature [24]. Furthermore, PCLM is often a later-stage event [13]. With the discovery of new survival-prolonging therapies, more PC patients are living longer to reach these later disease stages [13]. Therefore, it is expected that more PC patients will develop LM [13]. In fact, using the Surveillance, Epidemiology, and End Results (SEER) database, Kadeerhan et al. found an annual incidence rate increase of 12.3% of visceral metastasis (VM) in men with prostate cancer from 2010 to 2019 [25]. Moreover, Table 1 summarizes the rising rates of VM (including LM) reported in select phase III PC trials as a function of an increasing number of previous lines of therapy.

The limited existing research on PCLM as a unique metastatic site, as well as its poor prognosis, difficulties in early detection, and expected increasing incidence warrant further research. The present manuscript aims to provide a narrative review of the existing literature surrounding the circumstances of PCLM development, including patient demographics, clinical characteristics, the nature of the prior therapies received, and the associated biomarkers. Furthermore, through this review, we aim to identify areas of unmet need for further research.

## 2. Materials and Methods

### 2.1. Literature Search

A literature search was conducted through the Ovid Medline database to identify the literature regarding PCLM from 2000 until January 2024. Relevant subject headings and text words were used to capture the following three concepts: “Prostatic Neoplasms”, “Liver Neoplasms”, and “Neoplasm Metastasis”. The full search strategy is listed in Supplementary Table S1 (S1). Furthermore, backward citation searching of included articles was implemented to identify other studies of interest that were not identified in the initial literature search. No predefined limits on the study design or publication type were implemented. However, the present manuscript is focusing on LM in men with mCRPC (as opposed to very rare de novo LM in men with castration-sensitive prostate cancer).

### 2.2. Article Screening and Selection

**Figure 1:**
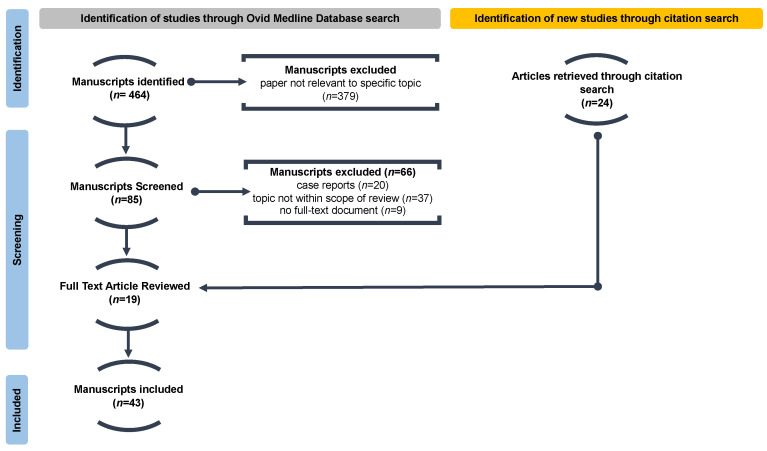
Consort diagram for inclusion/exclusion criteria. Four hundred and sixty-four manuscripts were identified through a search Query on Ovid Medline Database. Of these, 379 papers were not found to be relevant to the topic and were excluded. Next, an additional 66 manuscripts were removed for the following reasons: case reports (*n* = 20), topic was not within the review scope (*n* = 37), and no full-text documents (*n* = 9). The remaining 19 articles were thoroughly reviewed for eligibility, and an additional 24 manuscripts identified through citation searching were included to form a total of 43 studies in this review.

Article screening and selection were facilitated through the review manager Covidence, and the process is outlined in Figure 1 [34]. Four hundred and sixty-four manuscripts were identified through the database search. Of these, 379 articles did not relate specifically to PCLM and were subsequently excluded following title/abstract screening. Following, 85 papers were thoroughly reviewed. Of these, 66 were excluded for the following reasons: study outcomes were not specific to the onset of PCLM (*n* = 37), case studies (*n* = 20), and no full paper version was publicly available (*n* = 9). After this, only 19 studies remained. An additional 24 studies were identified through citation searching, with a total of 43 studies forming the basis for the present analysis.

## 3. Results

Prostate cancer is classified as castration sensitive or resistant. Metastatic castration-sensitive PC (mCSPC) can manifest at the initial time of diagnosis (de novo) or following a primary curative treatment attempt (metachronous) [35]. Of the 5–10% of PC patients in industrialized countries who present with de novo disease, 1.8% have been found to have LM [2,3,36]. Moreover, only 1.3–5% of men with mCSPC exhibit metachronous LM [37]. Thus, the majority of PCLM patients have mCRPC. While the mechanisms and risk factors for the emergence of LM in mCRPC remain poorly understood, the following have been described.

### 3.1. Clinicopathological Characteristics Associated with Liver Metastasis

Univariate analysis found that patients diagnosed with PC under the age of 70 years, with a tumor stage of ≥T3, locoregional lymph node involvement (N1) or de novo distant metastasis, a prostate specific antigen (PSA) greater than 20 ng/mL, or a Gleason score >8 were at increased risk for developing visceral metastasis (VM) [38]. Furthermore, VM has been found to be associated with concurrent nodal and bone metastases [13,39,40]. Alshalalfa et al. (2022) found no association between race (White vs. Black) and the site of mCRPC (*p* = 0.52) [17]. Interestingly, however, Akinyemiju et al. described 83% higher odds for developing de novo LM in non-hispanic (NH) Blacks with mCSPC (OR: 1.83, 95% CI) compared to NH-Whites [41].

### 3.2. Treatment-Emergent Prostate Cancer Liver Metastasis

#### 3.2.1. Androgen-Deprivation Therapy, including First Generation Anti-Androgens

The prostate is an androgen-dependent gland, requiring the binding of testosterone and dihydrotestosterone (DHT) to activate the androgen receptor (AR), an essential transcription factor for prostate morphogenesis and normal physiology [42]. PCs most commonly are AR-expressing adenocarcinomas whereby an altered AR-driven transcriptional program results in malignant features [43,44]. Thus, once primary curative treatments such as radiotherapy or radical prostatectomy have failed, the main line of PC treatment is androgen deprivation therapy (ADT) [45]. Aside from surgical castration, a hypogonadal state can be achieved by a class of drugs that act upon the luteinizing hormone-releasing hormone (LHRH, also known as the gonadotropin-releasing hormone [GnRH]) receptors to supress testosterone production by the testes through two distinct mechanisms [46]: (i) LHRH antagonists (e.g., degarelix, relugolix) competitively bind to LHRH receptors, inhibiting the downstream signaling of LH and thereby suppressing testosterone secretion [45]; and (ii) LHRH agonists (e.g., goserelin, leuprolide, and triptorelin), the most commonly used form of ADT, work by stimulating the LHRH receptors, creating an initial temporary surge in LH and testosterone, followed by eventual LH downregulation [45].

In contrast to ADT, which works to reduce the serum levels of testosterone, anti-androgens (AA), also referred to as AR antagonists, inhibit the binding of DHT and other androgens to the AR. First-generation oral nonsteroidal AA (e.g., Bicalutamide, Flutamide, and Nilutamide) may be prescribed in addition to ADT for testosterone surge protection, for a complete androgen blockade (CAB) or as a monotherapy [45,47].

Under prolonged treatment with ADT, AA, or CAB, PC cells eventually undergo a variety of mechanisms resulting in therapeutic resistance, i.e., CRPC; mCRPC is an incurable state of disease [14,42]. The progression to castration resistance has been found to significantly increase the rate of non-lung visceral metastasis, particularly to the liver [38].

#### 3.2.2. Second-Generation Anti-Androgens

In contrast to prior beliefs that CRPC was no longer androgen dependent, within the past two decades, AR signaling has been found to persist in CRPC through several mechanisms, including intratumoral androgen synthesis that is not suppressed by ADT [6,42]. This discovery led to the development of increasingly efficacious and potent second-generation AAs, which have largely replaced the first-generation AA and emerged as standard of care treatment options for CRPC patients [42]. Enzalutamide, Apalutamide, and Darolutamide are nonsteroidal competitive inhibitors of the AR, whereas Abiraterone is an androgen synthesis inhibitor [42]. Notably, Abiraterone and Enzalutamide are widely used to treat mCRPC [48].

A recent study by Iwamoto et al. found that the prior use of Abiraterone or Enzalutamide was associated with VM, particularly LM, in mCRPC patients [38]. While these drugs initially have a profound effect in slowing AR-driven tumor progression in most patients, the antitumor effects are often short-lived, and resistance eventually occurs [49,50]. Around 15–20% of CPRC adenocarcinomas eventually lose all AR dependence and undergo a transformation to AR-negative, poorly differentiated neuroendocrine PC (NEPC) [17,43]. Neuroendocrine differentiation remains vaguely understood, although it arises most commonly during the later stages of PC, driven by treatment-associated selective pressure [43,51]. Clinical evidence suggests that second-generation AAs can induce neuroendocrine features [52]. NEPC encompasses PC cells that display neuronal, endocrine, or a combination of both features; these cells can produce and secrete a variety of factors commonly found in the nervous system that stimulate tumor growth and, therefore, possess a very poor prognosis [22,43,53]. Many studies report that NEPC is closely associated with and commonly found in PCLM [16,17,22,53,54]. Since the increased use of Abiraterone and Enzalutamide is seen to increase the future incidence of LM as well, it is assumed that after the prolonged use of these drugs, PC cells gain resistance, and subsequent neuroendocrine transformation has occurred [38]. Of note, not all analyses discovered an independent association between Abiraterone or Enzalutamide and PCLM [13,55].

#### 3.2.3. Taxane Chemotherapy

Once the second-generation AA has failed, Docetaxel, a microtubule inhibitor of the taxane family, is typically the next line of mCRPC therapy [56,57]. However, most patients will become resistant over time, signifying further cancer progression and the activation of PC cell pro-survival pathways [56,57]. In the past decade, the FDA has approved the administration of Cabazitaxel, a second-generation taxane-based chemotherapy, to treat PC resistant to Docetaxel [56,58]. The interference with AR nuclear translocation is a recently discovered collateral consequence of the anti-microtubule properties of taxanes [59].

As with Abiraterone and Enzalutamide, the incidence of new VM, namely LM, has been found to increase significantly with the number of prior taxane chemotherapy treatments prescribed [22,38]. Taxane-resistant PC cells have also been associated with neuroendocrine differentiation, as well as the upregulation of the CCL2-CCR2 axis, which stimulates cancer cell migration and favors cancer progression through recruiting immunosuppressive cells to the tumor microenvironment [38,58].

### 3.3. Treatment-Induced Liver Injury

Liver injury is an adverse effect of PC therapeutics, reported in as many as 9% of PC patients [54]. Its cause is thought to be related to the liver’s responsiveness to androgens as liver cells express AR [54]. Liver AR expression has been implicated in several processes such as cellular metabolism, notably glycolysis, the infiltration by cells of the immune system, and the secretion of various cytokines and growth factors [54]. However, it remains to be demonstrated in detail how these hepatic AR functions might support metastatic growth in PC patients. Aside from interfering with AR signaling, most PC therapeutics are metabolized in the liver, which may result in liver-damaging intermediates [54]. In response to the onset of hepatic injury, the liver initiates an inflammatory and fibrotic response, leading to a series of immune cell activities [54]. Liver damage in patients with pancreatic and colorectal cancer has been seen to prepare a favorable pre-metastatic niche for the seeding and growth of tumor cells [54,60,61,62]. While more research is necessary, it is hypothesized that PC drug-induced liver injury may similarly promote the liver as a favorable pre-metastatic niche [54].

The use of AAs (first and second generation) has been associated with an increase in serum liver enzymes, a sign of liver damage, such as Flutamide (62% of patients), and to a lesser extent, Bicalutamide (6% of patients) [63,64]. Amongst second-generation AAs, Abiraterone has been linked most closely to increases in serum liver enzymes [65,66]. While the mechanism of hepatic injury is unknown, it is thought to arise from Abiraterone’s inhibition of CYP17, or its metabolization in the liver by the cytochrome P450 system that may produce a toxic intermediate, as a by-product of its breakdown [66]. In a recent study of patients administered Abiraterone, 28% experienced adverse effects; of this group, 47.4% suffered from liver dysfunction as determined by elevated levels of serum alanine aminotransferase (ALT), aspartate aminotransferase (AST), and/or bilirubin [67]. Furthermore, another study found that 13% of patients administered Abiraterone had elevations in serum aminotransferase compared with 1–8% receiving a placebo or a comparator drug [66]. More specifically, 6% of the patients receiving Abiraterone had ALT levels five times greater than the upper limit of normal (ULN), compared to <1% in the placebo group [66]. Moreover, in a retrospective analysis of 25 patients experiencing 46 episodes of Abiraterone-related liver injury, the toxicity was of grade 1, 2, and 3 according to the National Cancer Institute Common Terminology Criteria for Adverse Events (CTCAE) 4.03 in 7 (32%), 6 (27%), and 9 (41%) patients for ALT, and in 12 (50%), 6 (25%), and 6 (25%) for AST, respectively [65]. Only one patient had a concurrent transient bilirubin increase [65]. Overall, the National Institute of Diabetes and Digestive and Kidney Diseases (NIDDK) has rated Abiraterone a “C” on the likelihood scale of developing a clinically apparent liver injury, corresponding with a “probable rare cause of clinically apparent liver injury” [66]. Conversely, Apalutamide, Enzalutamide, and Darolutamide have all been ranked an “E” on the likelihood scale, pertaining to an “unlikely cause of clinically apparent liver injury”, and only rare cases of a clinically apparent liver injury with jaundice have been reported [68,69,70].

Up to 50% of patients administered Docetaxel have been seen to have elevated aminotransferase levels, although less than 2% had values greater than five times the upper limit of normal (ULN), and a clinically apparent liver injury from Docetaxel is relatively rare [71]. Nonetheless, individual cases have experienced severe acute hepatic necrosis [71]. As such, the NIDDK has rated Docetaxel a “C” on the likelihood scale of developing a clinically apparent liver injury [71]. In contrast, Cabazitaxel has been less associated with major increases in aminotransferase levels or reports of apparent liver injury, although few reports of acute hypersensitivity reactions may have the potential to lead to acute hepatic necrosis, resulting in an NIDDK rating of “E” [72].

Besides direct treatment-related liver injury, PC therapeutics may induce liver damage also indirectly. A study by Gild et al. found that men who underwent ADT were more likely to be diagnosed with nonalcoholic fatty liver disease (HR = 1.54, 95% CI), liver cirrhosis (HR 1.35, 95% CI), liver necrosis (HR 1.41, 95% CI), and any liver disease (HR 1.47, 95% CI) [73]. It remains to be investigated whether the rates of these liver conditions increase in PC patients undergoing therapy with AAs or taxane chemotherapeutics.

### 3.4. Biomarkers

#### 3.4.1. Serum Markers

A PSA doubling time (PSADT) of <12 months or ≤7.5 months is seen to be an independent predictor for the risk of distant metastasis in PC [74]. Specifically, shorter PSADTs have been found to be significantly associated with bone and visceral metastases [75]. However, no research to date has investigated the relationship between PSADT and the appearance of LM.

In fact, only few studies have reported biomarkers associated with the presence of LM [76]. Cotogno et al. conducted a study including mCRPC patients from three clinical trials. Through multivariable analysis, they found that an increase in AST and lactate dehydrogenase (LDH) and decreased levels of hemoglobin (Hb) were significant predictors of LM [76]. Specifically, regardless of Hb levels, patients with abnormally elevated AST and LDH had a greater than 25% risk of having LM [76]. Moreover, patients with Hb levels less than 9.5 g/dL and either an elevated AST or LDH were also at 25% greater risk [76]. Similarly, of all the mCRPC patients treated with Enzalutamide in the PREVAIL trial, patients with LM had higher baseline levels of LDH, as well as alkaline phosphatase (ALP) and PSA [21]. These findings were corroborated in a study by Ranasinghe et al. that evaluated serum markers in 23 patients with PCLM, including PSA, alkaline phosphatase (ALP), AST, ALT, LDH, and albumin; through univariate analysis, all markers were significantly associated with liver lesion volume [77]. There was a significant negative correlation between Hb or albumin and liver lesion volume, whereas the other markers were positively associated with the burden of LM. Multivariate analysis identified AST and Hb as optimal predictors of increasing liver lesion volume [77]. In addition, a study by Ghedini et al. found a statistically significant difference in the PSA levels between patients with LM (9.4 ng/mL) versus those without (5.7 ng/mL) [39].

Carcinoembryonic antigen (CEA) is another serum marker previously described as a molecular surrogate of aggressive variants of PC [78,79]. In a novel study by Bray et al., they investigated the effects of elevated CEA as an independent clinical and prognostic marker in patients with CRPC [78]. The authors found a significant decrease in median survival (*p* < 0.0001) and a significant increase in visceral metastasis (*p* = 0.03) in CRPC patients with elevated CEA; the latter was solely attributed to the increase in LM (+18.4%, *p* = 0.02), as there were no significant changes in the rates of metastases in other visceral sites [78]. Furthermore, there was no significant difference in metastases to the bones or lymph nodes in patients with or without elevated CEA [78].

While an increase in the chromogranin-A (CgA) protein in PC patients has been found to suggest the progression and/or presence of NEPC, Ploussard et al. observed that CRPC patients with elevated CgA levels had a significantly increased risk of LM [80].

#### 3.4.2. Genetic Markers

Overall, cellular and molecular mechanisms underlying PCLM such as lineage plasticity are greatly under-researched [12]. Lineage plasticity occurs when cancer cells lose dependency on the therapeutic target they once required for their function [81,82]. This phenomenon is common in PC cells that become resistant to ADT, when the cells lose characteristic adenocarcinoma histology and adapt to express neuroendocrine features and reduced AR activity [81,82,83]. Tumor suppressors such as the retinoblastoma protein (RB1), tumor protein 53 (TP53), and phosphatase and tensin homolog deleted on chromosome 10 (PTEN) repress lineage plasticity, ADT resistance, and metastasis in PC; accordingly, these genes are often mutated or lost in ADT-recurrent PCs [81,82,83,84]. Interestingly, PCLM tissues have been found to be associated with a higher fraction of genomic alterations compared to bone (*p* < 0.0001), lymph node (*p* < 0.0001), lung (*p* = 0.0008), and other metastatic sites (*p* = 0.009) [17]. Building upon these findings, Liu et al. generated genetically modified mouse models (GEMMs) to visualize the resulting histological and lineage features. Two GEMMs were formed: rb1Δ/Δp53Δ/Δ and ptenΔ/Δp53Δ/Δ [12]. Unlike the ptenΔ/Δp53Δ/Δ GEMMs, the rb1Δ/Δp53Δ/Δ GEMMs were found to display prominent LM [12]. Furthermore, the rb1Δ/Δp53Δ/Δ GEMMs contained a high percentage of CgA, synaptophysin (SYPT), and/or neural cell adhesion molecule (N-CAM) positive cells, all molecular markers associated with neuroendocrine features [12,85,86].

Nguyen et al. analyzed genomic and clinical data from a pan-cancer cohort of 25,000 patients with metastatic disease, including 2172 PC patients, corroborating the preclinical findings by Liu et al. [11]. In fact, PCLM patients (15% of the entire PC sub-cohort) had a higher frequency of RB1 loss compared to patients without LM (10% vs. 3%, q < 0.001). Moreover, they found an increase in PTEN loss (30% vs. 11%, q < 0.001) and Adenomatous Polyposis Coli (APC) mutations (11% vs. 5%, q = 0.001), the latter being another tumor suppressor gene implicated in prostate carcinogenesis [11,87]. In another clinical analysis, Alshalalfa et al. (2022) found that compared to other sites of PC metastasis, PCLM was significantly associated with PTEN deletion (42% vs. 20%), as well as phosphatidylinositol-4,5-bisphosphate 3-kinase catalytic subunit beta (PIK3CB) amplification (8.2% vs. 0.9%) and myelocytomatosis oncogene (MYC) amplification (29.5% vs. 9.8%) [17]. In corroboration, a study by Jin et al. reported MYC amplification to be more common in the liver compared to all other mCRPC sites [88].

Furthermore, the re-expression of the E-cadherin tumor suppressor gene has also been found in PCLM [9,89]. Normally, E-cadherin plays a key role in suppressing cell invasion and dissemination in epithelial cells. However, in the process of metastasis, epithelial cells undergo epithelial-mesenchymal transition and E-cadherin is lost [89,90]. Subsequently, the cells can break away from the primary site and navigate through the bloodstream to a remote location; at this point, they undergo a reverse process called epithelial mesenchymal reverting transition to colonize at the distant site [9,89,90]. Interestingly, it has been found that the interaction between metastatic PC cells and hepatocytes leads to a unique re-expression of E-cadherin in the liver [9,89]. This re-expression has been found to activate pro-survival kinases, rendering the disseminated PC cells chemoresistant, another explanation for the poor prognosis of PCLM [9].

## 4. Discussion

LM is the most lethal site of prostate cancer spread while at the same time, the most understudied. In this narrative review, we have identified the scarce existing literature regarding the risk factors for PCLM, grouping our findings into key areas, encompassing patient demographics and clinicopathological characteristics, treatment of emergent PCLM, liver injury and toxicity, as well as serum and genetic biomarkers.

Amongst others, we have identified chronic liver injury as a potential enabler of PCLM. Beyond PC treatments, other mechanisms may induce liver toxicity or injury, including pre-existing liver conditions, hepatotoxic medications administered for non-oncological indications, or alcohol consumption. Since liver injury and damage may render the liver a favourable metastatic site in PC patients, the specific associations between nonalcoholic fatty liver disease, as well as hepatitis or cirrhosis, and PCLM warrant further research. Furthermore, future studies should investigate the potential relationships between lifestyle factors or patient comorbidities and PCLM.

Given the relative rarity of LM in all-comer PC patient populations and the distinct molecular features of PCLM compared to other metastatic sites, future clinical studies may consider focusing on PCLM patients as a distinct study population. Furthermore, the combination of LM’s inherent metastatic dormancy, coupled with the liver being an uncommon site for routine screening in PC patients, underscores the necessity for studies on the early diagnosis of PCLM [18]. Identifying patients at an increased risk for LM is expected to enable selective screening strategies in these patients, paving the way for early detection methods, before there is widespread liver disease. However, we cannot discard the notion that there might be a stochastic element involved in the development of PCLM.

To date, there are no standard treatments specifically for patients with PCLM, who generally derive little benefit from systemic therapies that are otherwise effective in non-LM mCRPC patients. For instance, a systematic review by Yanagisawa et al. highlights the results of select mCRPC clinical trials, such as PREVAIL (Enzalutamide), PROfound (Olaparib), and CARD (Cabazitaxel), which do not suggest an OS benefit for patients with VM [21,29,33,91]. Consequently, there is a growing interest in locally ablative therapies, notably in patients with limited LM. In one case study, a 77-year-old man with PCLM treated with a combination of stereotactic image-guided percutaneous microwave ablation and Olaparib sustained remission from LM [92]. Furthermore, in another case study, a 66-year-old man with PCLM treated with radiofrequency ablation to the liver had no LM recurrence for 42 months [93]. Additionally, Yeo et al. showed the potential for microwave needle ablation and SBRT to target LM [94]. While these case studies demonstrate promise, they need validation in prospective randomized trials.

There are various limitations of this narrative review that must be acknowledged. As PCLM is an under-researched topic, the number of studies analyzed and included in this study were limited, potentially introducing bias into the findings. Furthermore, the combination of PC liver and lung metastases under the umbrella term “visceral metastases” in several studies limits the specificity of the results to PCLM. Nonetheless, this review serves as a valuable foundation for identifying gaps in knowledge while highlighting areas for future research regarding PCLM.

As PC patients continue to live longer due to an increasing number of life-prolonging therapies, it is anticipated that the incidence of patients with LM will increase. Hence, further research on the predictive factors of PCLM, as well as early detection methods and targeted therapies, is critical.

## Figures and Tables

**Table 1:** Percent of castration-resistant prostate cancer patients with visceral metastasis across phase III clinical trials. Trials were grouped based on previous treatment exposure of the population. Abbreviations: ADT, androgen deprivation therapy; AR, androgen receptor; CRPC, castration-resistant prostate cancer; LM, liver metastases; NHA, novel hormonal agent; NR, not reported; VM, visceral metastases.

1st line treatment	CRPC (i.e., resistant to ADT + 1st Generation AR Inhibitors)	Study	PREVAIL/ Enzalutamide [26] (Beer et al., 2014)	COU-AA-302/ Abiraterone [27] (Ryan et al., 2013)	
		% VM % LM	12% 4%	patients with VM excluded NR	
2nd line treatment	CRPC + Resistant to NHA	Study	PSMAfore/ ^177^Lu-PSMA-617 [28] (Sartor et al., ESMO 2023)	PROfound/ Olaparib [29,30] (de Bono et al. and Hussain et al., 2020) *	
		% VM % LM	NR 4%	32% NR	
2nd line treatment	CRPC + Chemoresistant (but no exposure to NHA)	Study	AFFIRM/ Enzalutamide [31] (Scher et al., 2012)	COU-AA-301/ Abiraterone [32] (de Bono et al., 2012)	
		% VM % LM	23% 10%	17.5% 10%	
3rd line treatment	CRPC + Chemoresistant + Resistant to NHA	Study	CARD/ Cabazitaxel [33] (de Wit et al., 2019)	VISION/ ^177^Lu-PSMA-617 [8] (Sartor et al., 2021)	PROfound/ Olaparib [29,30] (de Bono et al. and Hussain et al., 2020) *
		% VM % LM	18% 12%	21% NR	32% NR

* Study involved patients both pre- and post-Docetaxel chemotherapy treatment.

## Data Availability

No new data were created or analyzed in this study. Data sharing is not applicable to this article.

## Supplementary Materials

The following supporting information can be downloaded at: https://www.mdpi.com/article/10.3390/jcm13030734/s1, Table S1. Literature Search Strategy through Ovid Medline.
