# Supplementary material for: Prostate Cancer Liver Metastasis: An Ominous Metastatic Site in Need of Distinct Management Strategies
Source: J Clin Med. 2024 Jan 27;13(3):734. doi: 10.3390/jcm13030734 (PMC10856097; doi:10.3390/jcm13030734)
Supplement: Supplementary file 1 [file jcm-13-00734-s001.zip › jcm-2814197-supplementary.pdf]

Supplementary Materials:

Table S1. Literature Search Strategy through Ovid Medline.

#	Query	Results from 9 Feb 2023
1	exp Prostatic Neoplasms/	146,275
2	(prostat* adj3 (cancer* or neoplas* or tumo?r* or malignan* or carcinoma* or adenocarcinoma*)).ti,ab,kf,jw.	171,850
3	or/1-2 [Prostate Cancer]	194,162
4	exp Liver Neoplasms/	189,788
5	((liver or hepat*) adj3 (metast* or cancer* or neoplas* or tumo?r* or malignan* or carcinoma* or adenocarcinoma* or secondary)).ti,ab,kf,jw.	187,059
6	or/4-5 [Liver Cancer]	261,131
7	Neoplasm Metastasis/	113,592
8	metast*.ti,ab,kf.	613,273
9	(secondary adj3 (cancer* or neoplas* or tumo?r* or malignan* or carcinoma* or adenocarcinoma*)).ti,ab,kf.	21,423
10	or/7-9 [Metastases - Broad]	660,840
**11**	**3 and 6 and 10 [Broad option]**	**957**
12	(metast* adj3 (liver or hepat* or prostat*)).ti,ab,kf.	66,626
13	7 or 9 or 12 [Metastases – Specific]	190,628
**14**	**3 and 6 and 13 [Specific option]**	**552**
15	limit 14 to yr="2000 -Current"	464
